# Papillary thyroid carcinoma with pleomorphic tumor giant cells in a pregnant woman – a case report

**DOI:** 10.1186/s12902-018-0275-x

**Published:** 2018-07-05

**Authors:** Johan O. Paulsson, Jan Zedenius, C. Christofer Juhlin

**Affiliations:** 1 Department of Oncology-Pathology, Karolinska Institutet, Stockholm, Sweden; 2 Department of Molecular Medicine and Surgery, Karolinska Institutet, Stockholm, Sweden; 3 Department of Breast and Endocrine Surgery, Karolinska University Hospital, Stockholm, Sweden; 4 Department of Pathology and Cytology, Karolinska University Hospital, Stockholm, Sweden

**Keywords:** Papillary thyroid cancer, Pleomorphism, Pathology, Anaplastic thyroid cancer, Molecular testing

## Abstract

**Background:**

Papillary thyroid carcinoma with pleomorphic tumor giant cells (PTC-PC) is characterized by the occurrence of bizarre, pleomorphic cells within a small area of a conventional PTC. The histologic distinction between PTC-PC and PTC’s with a focal anaplastic thyroid cancer (ATC) component (denoted in the 2004 WHO classification as “papillary thyroid carcinoma with spindle and giant cell carcinoma”, PTC-SGC) is debated, however the prognosis is thought to be different (excellent for PTC-PC, poor for PTC-SGC). Therefore, this diagnostic challenge is significant for any endocrine pathologist to recognize. Herein, we report the histological and clinical workup of a PTC-PC case, with particular focus on the molecular analyses that facilitated the establishment of the final diagnosis.

**Case presentation:**

The patient was a pregnant, 28-year-old female presenting with a 30 mm conventional PTC, with focal areas with undifferentiated cells exhibiting exaggerated nuclear pleomorphism. No foci of extrathyroidal extension, angioinvasion or lymph node engagement were seen. Immunohistochemical analyses revealed the pleomorphic cells exhibiting retained differentiation. Molecular genetic analyses demonstrated a codon V600 missense mutation of the *BRAF* gene, but no *TP53* or *TERT* promoter mutations. The absence of an aggressive phenotype in addition to the lack of mutations in two major ATC-related genes led to the diagnosis of a PTC-PC. Postoperative MRI showed no evidence of metastatic disease. Radioiodine ablation was performed seven months post-operatively, and a SPECT-CT imaging did not show signs of residual tissue. She is well and without signs of disease 16 months post-operatively.

**Conclusions:**

PTC-PC is a differential diagnosis to PTC-SGC that mandates careful considerations. Taken together with previous publications, PTC-PC seems to be histologically similar to PTC-SGC, but clinically distinct. Even so, the distinction is not easily made given the different therapeutic consequences for each individual patient. This is the first report that includes molecular genetics to aid in finalizing the diagnosis. Exclusion of mutations in *TP53* and the *TERT* promoter could be considered as an adjunct tool when assessing papillary thyroid cancer with focal pleomorphism.

## Background

When evaluating thyroid tumors in the microscope, exaggerated pleomorphic cells and elevated Ki67-labeling index constitute two alarming histological features. These factors are almost always present in anaplastic (undifferentiated) thyroid cancer (ATC), but very seldom in papillary thyroid cancer (PTC) [1]. No clear guidelines in how to interpret focal areas with dedifferentiation have been established, as these tumors are so rarely encountered in clinical practice. Two different histological entities associated to pleomorphism (with great disparities in overall prognosis and outcomes) exist; namely papillary thyroid carcinoma with pleomorphic tumor giant cells (PTC-PC) and PTC’s with a focal anaplastic thyroid cancer (ATC) component (entitled “papillary thyroid carcinoma with spindle and giant cell carcinoma”, PTC-SGC) [1]. While PTC-PC cases generally display excellent prognosis, the survival rates for PTC-SGC are much poorer and often coupled to the extension of the anaplastic component. Therefore, the pleomorphic features in PTC-PC are not thought to be a result from a dedifferentiation to anaplastic carcinoma, as opposed to PTC-SGC, in which the focal pleomorphism constitute a bona fide ATC component [1].

In this case report, we depict a case of PTC with a focal pleomorphic component and how different clinical, histological and immunhistochemical analyses were used in the workup of this patient. We also describe for the first time how molecular testing steered the final histopathological diagnosis towards PTC-PC.

## Case presentation

The patient was a pregnant, 28-year-old female of Swedish ethnicity with no previous medical conditions or familial history, who experienced a lump in the neck during the summer of 2016. Physical examination was normal apart from the neck tumor. She did not exhibit any signs of dysphagia, hoarseness or discomfort. A 30 mm nodule was visible on neck ultrasound, and a first round of cytology was inconclusive (Bethesda I). A second round of cytology was performed, and a diagnosis of papillary thyroid cancer (Bethesda VI) was put forward based on the findings of follicular epithelium with nuclear atypia, nuclear inclusions and nuclear grooves. The tumor cells were positive for CK19 and HBME1, and the cytological Ki-67-index was estimated as 3–5%. Pleomorphic giant cells were not reported. Subsequent ultrasonographical mapping revealed no evident lateral lymph node engagements, and a total thyroidectomy with an associated lymph node dissection of regio VI was performed. The operation was carried out during the 2nd trimester and was uneventful without any postoperative complications.

**Fig. 1:**
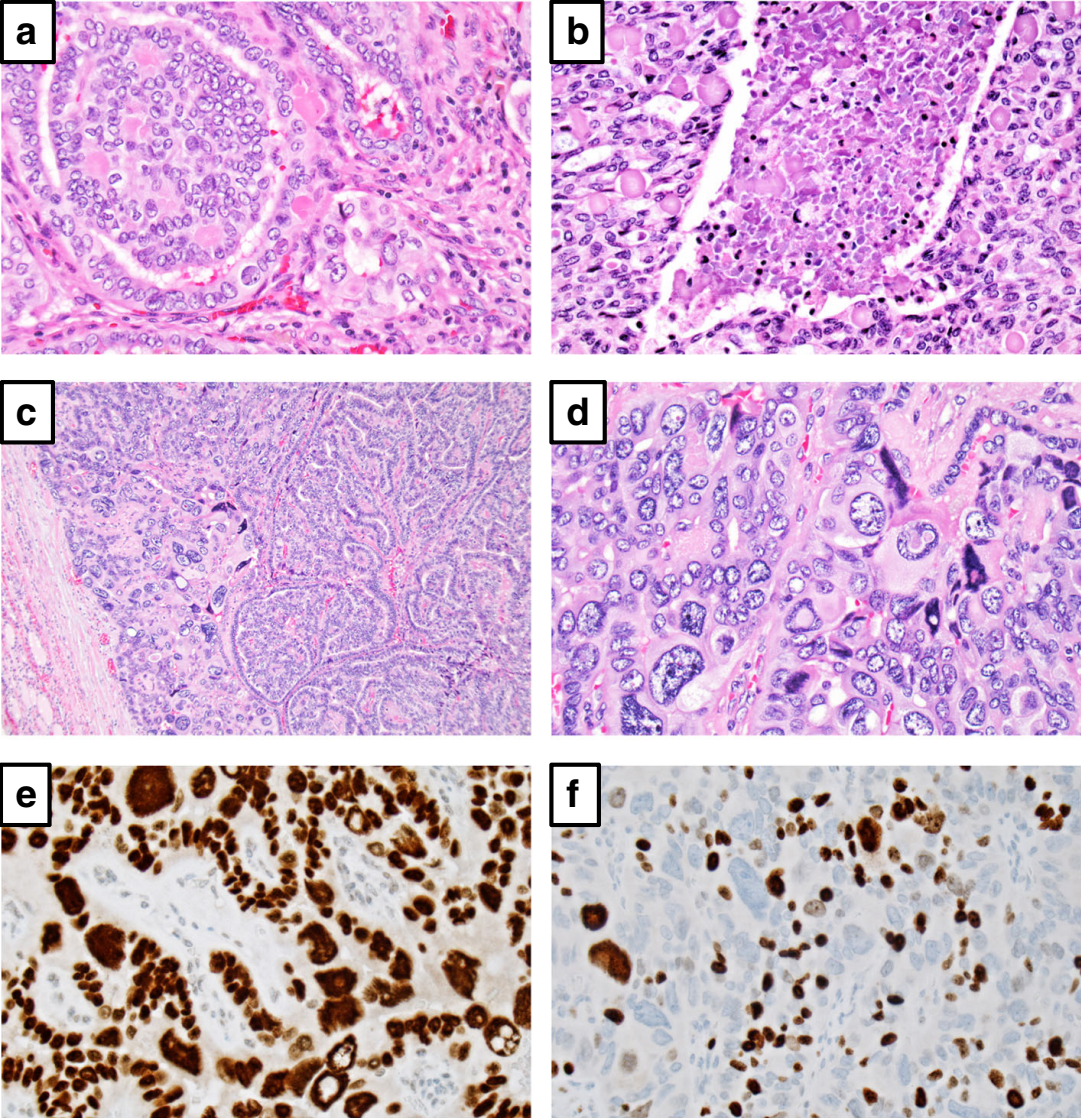
Photomicrographs of the PTC-PC case. **a** Routine sections of the conventional PTC area demonstrate clear-cut PTC-related nuclear features. **b** Focal tumor necrosis (central) within the conventional PTC area (left and right). **c-d** Focal areas with exaggerated pleomorphism and bizarre giant cells at 100× and 400× magnifications respectively. **e** Retained TTF1 immunoreactivity within the pleomorphic areas. **f** Markedly increased Ki67-labeling index within the same area (30% positive nuclei using immunohistochemistry with an anti-Ki-67 antibody)

The thyroid specimen exhibited a weight of 33,1 g. In the right lobe, a 30 × 30 × 25 mm well-defined nodule with firm, white to gray cut surface was visualized during macroscopic grossing. No macroscopic evidence of additional nodules were found in the isthmus or left thyroid lobe. Microscopy revealed a partly encapsulated, infiltrating tumor with a predominant papillary growth pattern, in addition to areas with follicular and solid growth patterns. Within most of the tumor area, the tumor cell nuclei were medium-sized, oval and exhibited a light chromatin, in addition to nuclear pseudo-inclusions and nuclear grooves (Fig. 1a). A few psammoma bodies were also seen scattered across the tumor area. This histological phenotype is consistent with a conventional papillary thyroid carcinoma (PTC) [1]. No extrathyroidal extension or foci with angioinvasion were seen. In the solid areas (constituting less than 10% of the tumor), PTC-like nuclear changes were more infrequent, but still present in small subsets of nuclei. Multifocally, areas with tumor necrosis (Fig. 1b) and elevated mitosis counts (5 mitoses/10 high power fields, but no atypical mitoses) were observed – but since the vast majority of the nuclei carried PTC-associated features (inclusions, grooves) in addition to the fact that the predominant growth pattern was papillary and no blood vessel infiltration was seen, no clear-cut diagnosis of poorly differentiated thyroid carcinoma (PDTC) could be made either by the older 2004 WHO criteria [1] or the proposed Turin criteria [2] supported by the novel 2017 WHO classification of tumors of endocrine organs [3]. The observed necrosis was believed to be tumor-related, as it was multifocal and without other degenerative changes usually associated to previous cytology aspiration.

In several foci near the tumor capsule, pleomorphic tumor cells with bizarre features were observed, including scattered giant cells (Fig. 1c and d). Strikingly, many of these giant nuclei exhibited prominent pseudo-inclusions, and a few had smudged chromatin. These cells were intermingled with more normal-sized tumor nuclei. The single, largest area with pleomorphic cells was 6 mm. The mitotic count was 5 mitoses/10 high power fields.

A tumor-free lymph node was found near the thyroid capsule, and an additional six tumor-free lymph nodes were visualized from the central compartment. As of this, no local metastatic disease could be found.

Immunhistochemical analyses were performed, and the tumor cells were uniformly positive for TTF1 (Fig. 1e), CK19 and Bcl-2. The cells were negative for chromogranin A and calcitonin, thereby excluding medullary thyroid cancer. Approximately 25% of all tumor cells expressed thyroglobulin. The p53 staining was weak and not diffuse, arguing against an underlying *TP53* mutation. A positive signal was obtained using the V600E mutation-specific BRAF antibody, arguing in favor for an underlying *BRAF* V600E missense mutation. There were no differences in the immunohistochemical profile when comparing the pleomorphic areas to the more conventional PTC areas, except for the Ki-67 proliferation labeling index, which was 10% in the conventional areas and up to 30% in the pleomorphic areas (Fig. 1f).

Although the immunohistochemical profile suggested retained differentiation (TTF1 positivity) and thus supported the diagnosis of PTC-PC [4], the focally observed high-proliferative areas with aggravated pleomorphism and bizarre nuclei led to the suspicion of tumor dedifferentiation into a local ATC component, consistent with PTC-SGC [1]. To better try to distinguish the two entities, a microdissection of formalin-fixated paraffin-embedded material representing the largest pleomorphic areas was performed. Tumor cell content was appreciated as 80%. Tissue was then deparaffinized and genomic DNA was extracted by established methods used in the clinical routine (Maxwell 16 FFPE Plus LEV DNA Purification Kit, Promega, WI, USA). Quality and quantity of genomic DNA was established using Nanodrop technology (NanoDrop Technologies). After DNA extraction, a control section was obtained and showed adequate representation of tumor tissue.

Three different genes were then assayed; *BRAF* (codon 600), *TP53* (exons 4–8) and *TERT* (promoter hotspot mutations C228T and C250T).

A 116 bp sequence of *BRAF* including codon 600 (part of exon 15) was amplified and analyzed using a real time PCR technique (Cobas 4800 *BRAF* V600 Mutation Test Kit, Roche Molecular Systems, NJ, USA). An activating mutation was found at codon V600. The test does not discriminate between V600E, V600 K and V600D.

For *TP53* and *TERT*, bi-directional Sanger sequencing was performed using conventional protocols (Genetic Analyzer 3500, Applied Biosystems, CA, USA). No *TP53* mutation in exons 4–8 was found, however, a known *TP53* single nucleotide polymorphism (SNP) in exon 4 was observed (c.215 C > G, p. P72R, rs1042522). This SNP has a reported minor allele frequency of 25,5% in the European American population according to the Exome Variant Server (http://evs.gs.washington.edu/EVS). Moreover, no C228T or C250T *TERT* promoter mutations were detected.

The case was presented at a multidisciplinary tumor board meeting held weekly at the Karolinska University Hospital. If the conclusive diagnosis would have been PTC-SGC, the patient would have continued with a much more aggressive and urgent treatment which would have required to abort the fetus, however with regards to the histopathology and the molecular profile of the tumor, a diagnosis of PTC-PC was argued for. But given the uncertainty of the histology as well as the high Ki67-labeling index, the patient was referred for magnetic resonance imaging (MRI) of the neck and chest, which was negative for pulmonary involvement, however displayed enlarged left-sided cervical lymph nodes in regio 2. Using ultrasonographic mapping, a fine needle biopsy was performed. The subsequent cytological examination however, could not detect any malignant cells.

Postoperative radioiodine ablation using 5400 MBq was performed six weeks after childbirth, seven months post-operatively. Whole body scintigraphy showed uptake in the neck, but a subsequent SPECT-CT did not show any sign of residual tissue. Serum thyroglobulin during stimulation by recombinant TSH was 2.1 micrograms/L. Basal thyroglobulin was 0.2 micrograms/L, without detectable thyroglobulin antibodies. Today, 16 months after surgery, the woman is well with no biochemical signs of disease.

## Discussion and conclusions

In the final pathology report, two different possibilities are discussed. Either the tumor represents a papillary thyroid carcinoma with pleomorphic tumor giant cells (PTC-PC), or the local finding of high-proliferative bizarre cells represents dedifferentiation into a focal anaplastic component (PTC-SGC). The WHO classification from 2004 [1] “rule-in” criteria for PTC-SGC is a focal undifferentiated component in minority compared to the PTC component, and hence this tumor could in theory be classified as such based on the focal findings of exaggerated pleomorphism. Indeed, the disturbingly high Ki67-labeling index within this area (30%) supports the notion of this minor component as highly aggressive. On the other hand, many factors argue in favor of the alterative diagnosis of PTC-PC. For example, the age of the patient and the clinical presentation is not typical for a focal ATC component. Moreover, the pleomorphic cells exhibited strong TTF1 and CK19 expression, which is often lost in ATCs [1]. Moreover, no histological signs of aggressive behavior existed, as the current case lacked extrathyroidal extension, an angio-invasive component and lymph node involvement [1, 4].

The molecular genetics in this case could pinpoint a codon V600 missense mutation in the *BRAF* gene, thereby proving the tumor as PTC-related [5]. However, since a large subset of ATCs de-differentiate from pre-existing PTCs, this marker does not exclude the occurrence of a synchronous ATC [6]. *TP53* mutations are seen in 50–80% of ATC cases but are rare in PTCs [7], and *TERT* promoter mutations are detected in 50–95% of ATC cases, but only in 10% of PTCs [8]. Interestingly, when co-occurring in the same patient, PTC and ATC cases display very high frequencies of *TERT* promoter mutations; 91 and 95% respectively [9]. The lack of *TP53* and *TERT* promoter mutations in our case could not entirely rule out ATC, but certainly not rule in ATC either. Therefore, the molecular genetics could not pinpoint the diagnosis, however speaks in favor of a PTC-PC diagnosis. Additional candidate genes arguing for dedifferentiation exist but were not tested as a part of the clinical workup as they are not established in our clinical pathology laboratory from a methodological standpoint. It is also worth mentioning that *TERT* promoter mutations in well-differentiated forms of thyroid cancer (such as PTC) are heavily coupled to older patient age [8]. Therefore, the young age of our patient could in theory affect the result of the *TERT* promoter mutational screening – which should be considered when screening adolescents for these aberrations. However, as the reason for conducting this molecular analysis was to exclude an overt ATC component, we believe a positive outcome (i.e. finding of a *TERT* promoter mutation) in such a young patient would strongly point towards a more aggressive form of the disease and support a focal ATC component.

ATCs arise either de novo or from a preexisting well or poorly differentiated thyroid carcinoma [1]. Nevertheless, the ATC component could be in minority, with the clear majority of the tumor constituting a well or poorly differentiated thyroid carcinoma. When the well differentiated tumor is a PTC, this focal ATC variant is denoted PTC-SGC [1]. However, the distinction between this tumor entity and a clear-cut ATC diagnosis is not easily established, and the prognosis of patients diagnosed with PTC-SGC is not entirely characterized. In a few series, patients with PTC-SGC have prolonged survival rates as compared to classical ATC cases, in other publications however, the prognosis was equally grim for PTC-SGC as for ATC [10, 11].

In a previous study, the authors describe four cases of PTC-PC, in which the pleomorphic areas constituted 5 to 25% of the tumor [4]. These four patients (three females and one male with a mean age of 36 years) remained alive without disease following thyroidectomy and ^131^I ablation therapy (with a mean follow-up time of 52 months), which is in strong contrast to the course of disease in patients with ATC or PTC-SGC. The authors therefore conclude that, based on the clinical and prognostic features of these cases, the bizarre cells found within these four tumors do not represent dedifferentiation and progression to ATC, and also argues against the diagnosis of PTC-SGC. Our patient mirrors the four previously published cases in regards to that no recurrences have been reported postoperatively, and no signs of extrathyroidal extension or lymph node metastasis have been found.

Moreover, there might be histological differences separating PTC-PC from PTC-SGC tumors as well, in that no areas of necrosis or inflammation was seen within the pleomorphic areas within our case (as well as in the previous four reported cases). Usually, the ATC component of a PTC-SGC is usually seen with tumor necrosis, a prominent inflammatory response as well as areas of fibrosis and hemorrhage [1]. Moreover, the bizarre pleomorphism in our case could in theory occur as a degenerative phenomenon rather than a feature associated to an aggressive malignant behavior. Indeed, the 2017 WHO classification of tumors of endocrine organs now lists “follicular adenoma with bizarre nuclei” as an acknowledged entity, thereby recognizing that well-differentiated thyroid tumors might exhibit highly atypical cell nuclei, similarly as PTC-PC cases.

We conclude that PTC-PC could constitute a separate histopathological variant of PTC, separate from PTC-SGC. PTC-PC should be considered as a differential diagnosis in younger patients when assessing PTCs with focal pleomorphism but retained differentiation (TTF1+, CK19+), no visible necrosis or inflammation and no obvious signs of extrathyroidal extension or lymph node involvement. As our final diagnosis relied heavily on the molecular testing, exclusion of mutations in *TP53* and the *TERT* promoter could be considered helpful when evaluating papillary thyroid cancer with focal pleomorphism.

## Abbreviations

ATC: Anaplastic thyroid cancer
bp: Base pairs
BRAF: B-Raf proto-oncogene, serine/threonine kinase
CK19: Cytokeratin 19
MBq: Megabecquerel
MRI: Magnetic resonance imaging
PCR: Polymerase chain reaction
PDTC: Poorly differentiated thyroid cancer
PTC: Papillary thyroid cancer
PTC-PC: Papillary thyroid carcinoma with pleomorphic tumor giant cells
PTC-SGC: Papillary thyroid carcinoma with spindle and giant cell carcinoma
SPECT-CT: Single-photon emission computed tomography
TERT: Telomerase reverse transcriptase
TSH: Thyroid stimulating hormone
TTF1: Thyroid transcription factor 1
